# Rapid Assessment of Avoidable Blindness in India

**DOI:** 10.1371/journal.pone.0002867

**Published:** 2008-08-06

**Authors:** John Neena, Jose Rachel, Vashist Praveen, Gudlavalleti V. S. Murthy, for the RAAB India Study Group

**Affiliations:** 1 Community Ophthalmology Unit, Dr. R. P. Centre for Ophthalmic Sciences, All India Institute of Medical Sciences, New Delhi, Delhi State, India; 2 Health Services, Ministry of Health & Family Welfare, Government of India, New Delhi, Delhi State, India; University of Cape Town, South Africa

## Abstract

**Background:**

Rapid assessment of avoidable blindness provides valid estimates in a short period of time to assess the magnitude and causes of avoidable blindness. The study determined magnitude and causes of avoidable blindness in India in 2007 among the 50+ population.

**Methods and Findings:**

Sixteen randomly selected districts where blindness surveys were undertaken 7 to 10 years earlier were identified for a follow up survey. Stratified cluster sampling was used and 25 clusters (20 rural and 5 urban) were randomly picked in each district.. After a random start, 100 individuals aged 50+ were enumerated and examined sequentially in each cluster. All those with presenting vision <6/18 were dilated and examined by an ophthalmologist. 42722 individuals aged > = 50 years were enumerated, and 94.7% examined. Based on presenting vision,, 4.4% (95% Confidence Interval[CI]: 4.1,4.8) were severely visually impaired (vision<6/60 to 3/60 in the better eye) and 3.6% (95% CI: 3.3,3.9) were blind (vision<3/60 in the better eye). Prevalence of low vision (<6/18 to 6/60 in the better eye) was 16.8% (95% CI: 16.0,17.5). Prevalence of blindness and severe visual impairment (<6/60 in the better eye) was higher among rural residents (8.2%; 95% CI: 7.9,8.6) compared to urban (7.1%; 95% CI: 5.0, 9.2), among females (9.2%; 95% CI: 8.6,9.8) compared to males (6.5%; 95% CI: 6.0,7.1) and people above 70 years (20.6%; 95% CI: 19.1,22.0) compared to people aged 50–54 years (1.3%; 95% CI: 1.1,1.6). Of all blindness, 88.2% was avoidable. of which 81.9% was due to cataract and 7.1% to uncorrected refractive errors/uncorrected aphakia.

**Conclusions:**

Cataract and refractive errors are major causes of blindness and low vision and control strategies should prioritize them. Most blindness and low vision burden is avoidable.

## Introduction

India was the first country in the world to initiate a public funded program for the control of blindness as a national priority health problem [1]. Since the inception of the program, harnessing evidence to support the program and to guide its implementation has been the key to identify effective strategies [2]. Population based surveys have been the main source for providing information on whether the program was progressing in the right direction [2]–[4]. These surveys have been undertaken at periodic intervals over the past two decades [2]–[4].

Rapid assessment of cataract blindness has been accepted as a robust tool to help planners in developing countries including India [5]–[8]. Initially, these techniques were limited to ascertaining cataract blindness, visual outcomes after cataract surgery, cataract surgical coverage and barriers to cataract surgery [9]–[14]. This was immensely important as cataract has been recognized as the commonest cause of blindness and severe visual impairment in India [1]–[4], [14]–[22].

With the launch of Vision 2020 global initiative the focus has shifted to all causes of avoidable blindness rather than being limited to cataract and rapid assessments have been expanded to include all causes of avoidable blindness [23]–[26]. Since this tool has been found to be valid, it was proposed to undertake a rapid assessment of avoidable blindness (RAAB) among people aged 50 years and above in India in 2007, in the most populated regions of the country to assess the changes in the prevalence of avoidable blindness.

## Methods

In 2007, RAAB was undertaken in 16 districts in 15 States in India. The districts identified were those where population based surveys for blindness was undertaken over the period 1998–2001. These districts were randomly selected during the earlier surveys. The same districts were identified to evaluate whether there was a difference in the prevalence since the last survey.

Stratified cluster random sampling was used for the survey. Separate sampling frames were constructed for two strata identified for the survey–rural and urban. The sampling universe consisted of all those aged 50+ years who were habitual residents (staying in the village for at least the previous 6 months).

Sample size was determined using a prevalence estimate of 10% for blindness and severe visual impairment among those aged 50 years and above. The World Health Organization (WHO) defines blindness as presenting vision <3/60 in the better eye and severe visual impairment as presenting vision <6/60–3/60 in the better eye while in India both categories are clubbed together to define blindness. Power of 80%, relative precision (error bound) of 20%, confidence level of 95% and design effect of 2 were used to calculate sample size. With this criteria, a sample size of 2500 per district spread over 25 clusters (20 rural and 5 urban) was covered.

A cluster was constituted by a population segment of 850–1000 people of all ages. Villages with smaller populations were clubbed together while villages with larger populations were segmented so as to yield clusters of equal sizes. Since 13% of the Indian population is aged 50 years or older [27], a cluster of 850–1000 population would yield 110–130 people aged 50 years or older. All the clusters together constituted the sampling frame. 35 clusters (20 rural and 5 urban) were then randomly picked using random number tables. A random starting point in each cluster was identified by first identifying the centre of the cluster by the enumeration team in consultation with the local leaders (both formal and non-formal). The number of lanes emanating from the central point was identified and the number put down on slips of paper. One slip was randomly picked up by the local leaders to identify the direction in which the enumeration team should proceed. Next the first occupied house on the right side of the lane was identified as the starting point in the cluster.

In each cluster, random walk method was used to enumerate and examine the first 100 people aged 50 years and more in each cluster, after identifying a random starting point in the cluster. The random walk method has been used in house-to-house surveys wherein after identifying a central location in a cluster, a random direction is chosen and after starting from a random household, the next nearest household is visited until the required sample has been assembled in the cluster [28].

Ethical approval was provided by the Government of India. Informed consent was obtained from all respondents in the presence of two responsible members of the local community.

Historical events were used to identify the age of the respondents. Individuals aged 50 years and above and resident in the cluster continuously for the past six months were eligible for inclusion. Informed verbal consent was obtained from each eligible individual, in the presence of a local witness and examination was only undertaken after consent was obtained.

A modified Early Treatment Diabetic Retinopathy Study (ETDRS) chart with 5 ‘Es’ corresponding to 6/18 on one side and another 5 ‘Es’ corresponding to 6/60 cut-off on the reverse side was used from a distance of 4 metres in shaded day light. Trained ophthalmic assistants administered the vision tests. All those individuals who could not identify 4 of the 5 ‘Es’ of the 6/18 optotypes were examined by an ophthalmologist. Vision was recorded separately for each eye, with any distance correction that the person was using (presenting vision). All individuals with vision <6/18 in any eye also had their vision recorded with a pinhole and the pinhole vision was recorded separately. If an individual could not read the 6/60 optotypes from a distance of 4 metres, their vision was tested again at a distance of 2 meters.

All individuals who failed to see the 6/18 optotypes from 4 meters with any eye were brought to a makeshift clinical station set up in the cluster for being examined by an ophthalmologist. Wherever the ophthalmologist felt that dilation was essential to make a diagnosis, the eyes were dilated.

A clinical algorithm was used to identify the most likely cause of impairment in each eye and the most ‘preventable’ or ‘currently treatable’ cause was given precedence over a non preventable or treatable cause. The same process was used for identifying the principal cause of blindness for the person. This is the procedure recommended by the WHO. The clinical algorithm was set out by a team of senior ophthalmologists through a process of consultation and consensus.

The following case definitions were used for identifying the cause of visual impairment:

Refractive error: Vision <6/18 improving to > = 6/18 with pin hole.

Cataract: Presence of a visible cataract impairing vision.

Glaucoma: In the absence of any other obvious cause, presence of significant pallor **AND** Cup: Disc (C: D) ratio >0.6 along with pigmentary changes and other signs of glaucoma including evidence of iridectomy/blebs etc and C: D asymmetry >0.2 between the two eyes.

Diabetic Retinopathy: Sight threatening retinopathy was to be allocated as the cause of visual impairment when:

- there were more than 5 microaneurysms.
- Clinically significant Macular Edema (CSME) on Distant Direct Ophthalmoscopy
- Neo Vascularisation of the Disc/Neo Vascularisation Elsewhere (NVD/NVE)
- Vitreous Haemorrhage

‘Suspected’ Age related macular degeneration (ARMD) was marked as the cause of visual impairment (VI) when:

- There was drusen at macula
- Macular scar was present.
- “Wet” ARMD was recorded
- Geographic atrophy was observed

## Results

A total of 42722 individuals were enumerated and 40447 (94.7%) were examined in 16 districts in 15 of the most populated States in the country. 78.5% of the enumerated resided in the rural areas (Table 1). A third (35.4%) were aged 65 years and above and the mean age of both the enumerated and the examined was 61.5 years. Nearly a fifth (18%) of the respondents was not engaged in any form of work. There were no significant differences between the enumerated and examined populations in relation to place of residence, gender, age groups or work status (Table 1). Census figures show that among those aged 50 years and older, 34.9% are 65 years and older [27]. Similarly among the 50 + population, 50.5% are male and 49.5% are female [27].

**Table 1:** Basic Demographic characteristics of the Surveyed Population.

Parameters	Enumerated		Examined	
	N	%	N	%
Total 50+ population	42722		40447	94.7
**Residence**				
Urban	9175	21.5	8625	21.3
Rural	33547	78.5	31822	78.7
**Gender**				
Male	19460	45.5	18181	45
Female	23262	54.5	22266	55.1
**Age Groups**				
50–54 yrs	9725	22.8	9388	23.2
55–59 yrs	9162	21.5	8639	21.4
60–64 yrs	8809	20.6	8205	20.3
65–69 yrs	6085	14.2	5738	14.2
> = 70 yrs	8941	20.9	8477	21
Mean age	61.5		61.5	
**Work status**				
Active work	19111	44.7	17932	44.3
House work	15687	36.7	15115	37.4
No work	7702	18	7279	18.0
No response	222	0.5	121	0.3

Among the examined population, 4.4% (95% CI: 4.1–4.8) were severely visually impaired (SVI) (<6/60–3/60 in the better eye on presenting vision) and 3.6% (95% CI: 3.3–3.9) were blind (vision<3/60 in the better eye on presenting vision) (Table 2). Additionally, 16.8% (95% CI: 16.0–17.5) suffered from (VI) (<6/18–6/60). The prevalence of VI, severe visual impairment (SVI) and blindness increased with age (Table 2). Similarly, women had a higher prevalence of VI, SVI and blindness compared to males as did rural residents considering SVI and blindness. Actively working individuals had significantly lower prevalence of VI, SVI and blindness compared to those not working (Table 2).

**Table 2:** Distribution of Vision Categories (Presenting Vision In better eye).

Parameters	Normal/Near Normal (> = 6/18)	Visual Impairment (<6/18–6/60)	Severe Visual Impairment (<6/60–3/60)	Blindness (<3/60)	Vision Not assessed
All	30413 (75.2%)	6786 (16.8%)	1797 (4.4%)	1443 (3.6%)	8 (0.02%)
(N:40447)	[95% CI: 74.2–76.2]	[95% CI: 16.0–17.5]	[95% CI:4.1–4.8]	[95% CI:3.3–3.9]	
**Age**					
50–54 y	8709 (92.8%)	555 (5.9%)	79 (0.8%)	44 (0.5%)	1 (0.01%)
(N:9388)	[91.9–93.7]	[5.1–6.8]	[0.7–1.0]	[0.3–0.6]	
55–59 y	7474 (86.5%)	922 (10.7%)	170 (2.0%)	70 (0.8%)	3 (0.03%)
(N:8639)	[85.6–87.5]	[9.7–11.6]	[1.6–2.3]	[0.6–1.0]	
60 64 y	6193 (75.5%)	1475 (18.0%)	342 (4.2%)	185 (2.4%)	0
(N:8205)	[74.1–76.9]	[16.9–19.0]	[3.5–4.9]	[2.0–2.8]	
65–69 y	3782 (65.9%)	1358 (23.7%)	361 (6.3%)	236 (4.1%)	1 (0.02%)
(N:5738)	[64.3–67.6]	[21.9–25.4]	[5.7–6.8]	[3.6–4.6]	
70 + y	4255 (50.2%)	2476 (29.2%)	845 (10.0%)	898 (10.6%)	3 (0.04%)
(N:8477)	[48.6–51.8]	[28.0–30.4]	[9.1–10.9]	[9.7–11.5]	
**Gender**					
Male	14038 (77.2%)	2950 (16.2%)	670 (3.7%)	520 (2.9%)	3 (0.02%)
(N:18181)	[76.1–78.4]	[15.4–17.0]	[3.3–4.1]	[2.5–3.2]	
Female	16375 (73.5%)	3836 (17.2%)	1127 (5.1%)	923 (4.1%)	5 (0.02%)
(N:22266)	[72.4–74.7]	[16.3–18.2]	[4.6–5.5]	[3.7–4.5]	
**Residence**					
Urban	6525 (75.6%)	1485 (17.2%)	360 (4.2%)	254 (2.9%)	1 (0.01%)
(N:8625)	[71.1–80.2]	[14.1–20.3]	[3.1–5.3]	[1.7–4.1]	
Rural	23888 (75.1%)	5301 (16.7%)	1437 (4.5%)	1189 (3.7%)	7 (0.02%)
(N:31822)	[74.2–76.0]	[15.9–17.4]	[4.2–4.9]	[3.4–4.0]	
**Work Status**					
Actively Working	15068 (84.0%)	2320 (12.9%)	380 (2.1%)	160 (0.9%)	4 (0.02%)
(N:17932)	[82.9–85.1]	[12.0–13.8]	1.7–2.5]	[0.7–1.1]	
House Work only	11868 (78.5%)	2387 (15.8%)	598 (4.0%)	261 (1.7%)	1 (0.01%)
(N:15115)	[77.3–79.7]	[14.8–16.8]	[3.6–4.3]	[1.4–2.0]	
No work	3379 (46.4%)	2067 (28.4%)	813 (11.2%)	1017 (14.0%)	3 (0.04%)
(N: 7279)	[44.7–48.2]	[27.3–29.5]	[10.2–12.1]	[12.8–15.1]	
No response	98 (81.0%)	12 (9.9%)	6 (5.0%)	5 (4.1%)	0
(N:121)					

Use of pin hole reduced the proportion of respondents who could be categorised as VI, SVI or blind compared to presenting vision (Table 3). Those with normal vision increased to 84.7% with pin hole compared to 75.2% with presenting vision. However the proportion of blind reduced to 3% from 3.6% meaning thereby that 83.6% of the blind did not improve with pinhole (Table 3).

**Table 3:** Comparison of presenting and pin-hole vision categories*.

Presenting Vision	Pin hole Vision				Total
	> = 6/18	<6/18–6/60	<6/60–3/60	<3/60	
> = 6/18	30413	-	-	-	30413 (75.2%)ˆ
<6/18–6/60	3666 (54.0%)	3120 (46.0%)	-	-	6786 (16.8%)ˆ
<6/60–3/60	146 (8.1%)	628 (34.9%)	1023 (56.9%)	-	1797 (4.4%)ˆ
<3/60	20 (1.4%)	73 (5.1%)	143 (1.0%)	1207 (83.6%)	1443 (3.6%)ˆ
Total	34245 (84.7%)	3821 (9.4%)	1166 (2.9%)	1207 (3.0%)	40439 (100%)

ˆ Column %.

* Vision could not be assessed in 8 persons.

The Indian definition of blindness is vision <6/60 in the better eye on presenting vision. This definition includes the category of SVI and blindness as defined by the WHO. Using the Indian definition, the overall prevalence of blindness (<6/60 in the better eye) was 8.0% (95% CI: 7.54–8.48) (Table 4). Compared to respondents aged 50–54 years, those aged 60–64 years had a 3.65 times higher risk while those aged 70 years and above had a 7.4 times higher risk of being blind. This difference was statistically significant (X^2^-755.14; p<0.0001). Females had a 56% higher risk of blindness compared to males (Adj. OR- 1.56; X^2^-111.06; p<0.0001). Rural respondents had a 1.2 times higher risk of being blind compared to the urban respondents (X^2^- 14.75; p<0.001)(Table 4). Those who were not engaged in any productive work had 4.2 times higher risk of being blind compared to those who were engaged in active productive work(Adj. OR- 4.18; X^2^-826.17; p<0.0001).

**Table 4:** Relationship of Blindness & SVI (Vision<6/60 presenting) with Socio-Demographic variables.

Parameter	Prevalence of Blindness	Adjusted OR*	95% CI	P
**All 50+**	8.0% [95% CI:7.54–8.48]			
**Residence**				
Urban	7.12%	1.0		
	[4.98–9.25]			
Rural	8.25%	1.21	1.1–1.33	X^2^-14.75; p<0.001
	[7.88–8.63]			
**Age Category**				
50–54 yrs	1.3%	1.0		
	[1.06–1.56]			
55–59 yrs	2.8%	1.91	1.54–2.38	X^2^-35.04; p<0.0001
	[2.40–3.15]			
60–64 yrs	6.5%	3.65	2.99–4.45	X^2^-187.82; p<0.0001
	[5.78–7.32]			
65–69 yrs	10.4%	4.92	4.03–6.01	X^2^-299.51; p<0.0001
	[9.78–11.03]			
> = 70 yrs	20.6%	7.42	6.07–9.06	X^2^-533.09; p<0.0001
	[19.14–21.98]			
X-755.14; p<0.0001				
**Gender**				
Male	6.54%	1.0		
	[6.19–6.90]			
Female	9.21%	1.56	1.45–1.72	X^2^-111.06; p<0.0001
	[8.83–9.59]			
X-111.06; p<0.0001				
**Work Category**				
Actively Working	3.01%	1.0		
	[2.76–3.26]			
House work	5.68%	1.33	1.17–1.52	X^2^-18.02; p<0.0001
	[5.17–6.20]			
No work	25.14%	4.18	3.7–4.72	X^2^-626.69; p<0.0001
	[23.56–26.72]			
X-826.17; p<0.0001				

* Adjusted for Age, Gender, Residence, Work Status.

Cataract was the commonest cause of overall blindness and avoidable blindness and VI (Table 5). Refractive errors including uncorrected aphakia were responsible for 6.3% of blindness while complications after cataract surgery were responsible for 3% of all blindness. Corneal opacities (including trachoma) were responsible for 6.5% of blindness while Glaucoma was responsible for 4.4% of blindness. Uncorrected refractive errors were responsible for a third (32.9%) of VI and were the second commonest cause after Cataract (58.1%) among those with VI. Among the blind, 88.2% were blind due to avoidable blindness while among VI, 96.4% was due to avoidable causes. Cataract was the single most important cause of all avoidable blindness and VI (Table 5). Using the Indian definition of blindness, cataract was responsible for 77.5% of all blindness and 84.4% of avoidable blindness. Refractive errors/uncorrected aphakia were responsible for 8.7% of blindness as per the Indian definition (Table 5).

**Table 5:** Causes of Blindness and Low Vision.

Cause	All						Avoidable					
	<6/18–6/60		<3/60		<6/60		<6/18–6/60		<3/60		<6/60	
	(N:6623)		(N:1436)		(N:3226)		(N:6384)		(N:1266)		(N:2962)	
	N	%	N	%	N	%	N	%	N	%	N	%
Refractive Errors	2177	32.9	9	0.7	109	3.4	2177	34.1	9	0.7	109	3.7
Cataract	3846	58.1	1037	72.2	2501	77.5	3846	60.1	1037	81.9	2501	84.4
Uncorrected Aphakia	177	2.7	81	5.6	148	4.6	177	2.8	81	6.4	148	5.0
Cataract Surgery Complications	103	1.6	43	3.0	70	2.2	103	1.6	43	3.4	70	2.4
Phthisis	5	0.05	25	1.7	27	0.8	NA					
Trachoma	3	0.03	9	0.6	10	0.3	3	0.05	9	0.7	10	0.3
Other corneal opacities	64	1	85	5.9	117	3.6	64	1.0	85	6.7	117	3.9
Globe abnormalities	1	0.02	7	0.5	8	0.2	NA					
Glaucoma	64	1	63	4.4	96	3	NA					
Diabetic Retinopathy	14	0.2	2	0.1	7	0.2	14	0.2	2	0.2	7	0.2
Suspected AMD	60	0.9	10	0.7	31	1	NA					
Other post segment	61	0.9	31	2.2	52	1.6	NA					
Other causes	43	0.7	26	1.8	39	1.2	NA					
Undetermined	5	0.08	8	0.6	11	0.3	NA					
Avoidable Causes	6384	96.4	1266	88.2	2962	91.8	NA					

## Discussion

The planning and implementation of appropriate eye care services in a country needs evidence generated from populations. Such exercises being costly have not been used extensively in many countries. Recently, rapid assessment techniques have been developed which provide valid estimates in a short period of time and also reduce the overall cost of conducting a survey [5]–[14], [23]–[26]. Rapid assessments have been used in India for more than 10 years and have provided the backdrop for district level planning of services [6], [7], [14].

With the clarion call for the elimination of avoidable blindness by 2020, rapid assessments have evolved to include all causes of avoidable blindness like cataract, refractive errors, trachoma and other causes of corneal scarring [25]. For the first time, the methodology was developed and used for rapid assessment of avoidable blindness in India.

The prevalence of blindness (vision<3/60 in the better eye-presenting vision) among those aged 50 years and over was 3.6%. This is significantly lower than the prevalence reported in a national survey over the period 1999–2001 where a detailed eye examination was undertaken (5.3%; 95% CI: 5.1–5.6) [2] and a rapid assessment in 1998 which covered most of the highly populated states in India (5.24%; 95% CI: 4.98–5.62)[14]. However the present estimates in India are much higher than what has been reported in China, East Africa and Bangladesh [23], [24], [26], [29]. This is despite WHO's conclusions that there has been a 25% reduction in the prevalence of blindness in India [30]. There is ample evidence in India that there is a meteoric increase in life expectancy and consequently the elderly populations in India [31], [32]. The sudden increase in the population above 50 years due to significant increases in life expectancy in India may be the most important reason for the much higher prevalence of blindness in the Indian sub continent compared to other countries which share similar geographical and topographical characteristics, even though the augmented service delivery networks in the country have helped in reducing the prevalence of blindness.

Gender disparities and poorer access to services in the rural areas are still a challenge in India as has been documented in the present study. Renewed efforts will be needed in this direction.

Cataract continues to be the commonest cause of blindness in India with three out of every four blind above the age of 50 years being blind due to cataract. This is higher than what has been reported from studies using a detailed eye examination in India earlier [2]. A rapid assessment in Bangladesh has also observed that cataract was responsible for 79% of blindness among those aged 50+ [27]. It is likely that the examination method used in rapid assessments represents an over-diagnosis of cataract because the posterior segment cannot be assessed as carefully as in a detailed examination and because a complete examination for glaucoma is not possible without assessing the visual fields. Another problem with rapid assessment as with other surveys is that the examination is done during the day light hours and working males would be underrepresented in the survey. This is also seen in the present study where though the census figures show that 49.5% of the population above 50 years is female[27], there were 54.5% of women enumerated in the RAAB.

Evidence from rapid assessments from the Indian sub continent compared with rapid assessments in other parts of the world reveals that cataract blindness is significantly commoner in South Asia [14], [29] compared to Africa [23], [26]. This may again be due to the recent significant increase in life expectancy in South Asia while the life expectancy in Africa has increased much more gradually. Adjusting for age structures in Africa and Asia would provide a better perspective in this regard. If there is indeed a higher prevalence of cataract blindness, then cataract would remain a priority for elimination of avoidable blindness in South Asia till service delivery matches the increased life expectancy of South Asian populations. It is therefore important that funding be committed for cataract surgical services in South Asia at least for the next two decades. Similarly refraction services need to be augmented as a significant proportion of VI is due to uncorrected refractive errors/uncorrected aphakia.

In India 91.8% of blindness among the 50+ is avoidable (refractive errors, cataract, surgical complications, aphakia, trachoma and corneal scars, diabetic retinopathy). This is higher than what has been reported in most other countries [23], [24], [26], [29]. This is again due to the high prevalence of cataract blindness in India. Evidence suggests that over the next decade if cataract surgical services and refraction services are augmented both in quantity and quality, the country would be well prepared to eliminate avoidable blindness by 2020. If this does not happen, then the likelihood of achieving the goals of Vision2020: The Right to Sight initiative would remain a dream that would take till eternity to be translated into reality.
